# Comparison of three DNA extraction methods for the detection and quantification of GMO in Ecuadorian manufactured food

**DOI:** 10.1186/s13104-017-3083-x

**Published:** 2017-12-20

**Authors:** Ricardo Pacheco Coello, Jorge Pestana Justo, Andrés Factos Mendoza, Efrén Santos Ordoñez

**Affiliations:** 1 ESPOL Polytechnic University, Escuela Superior Politécnica del Litoral, ESPOL, Centro de Investigaciones Biotecnológicas del Ecuador, Campus Gustavo Galindo, Km. 30.5 vía Perimetral, P.O. Box 09-01-5863, Guayaquil, Ecuador; 2 Agencia Nacional de Regulación, Control y Vigilancia Sanitaria, ARCSA, Ciudadela Samanes, Av. Francisco de Orellana y Av. Paseo del Parque, Bloque 5, Guayaquil, Ecuador; 3 Biosafety Unit, National Biodiversity Direction, Ministry of Environment, Madrid y Andalucía, Quito, Ecuador; 4 ESPOL Polytechnic University, Escuela Superior Politécnica del Litoral, ESPOL, Facultad de Ciencias de la Vida, Campus Gustavo Galindo, Km. 30.5 vía Perimetral, P.O. Box 09-01-5863 Guayaquil, Ecuador

**Keywords:** GMOs, PCR, DNA extraction, Processed food

## Abstract

**Objectives:**

In Ecuador, food products need to be labeled if exceeded 0.9% of transgenic content in whole products. For the detection of genetically modified organisms (GMOs), three DNA extraction methods were tested in 35 food products commercialized in Ecuador. Samples with positive amplification of endogenous genes were screened for the presence of the Cauliflower mosaic virus 35S-promoter (P35S) and the nopaline synthase-terminator (Tnos). TaqMan™ probes were used for determination of transgenic content of the GTS 40-3-2 and MON810 events through quantitative PCR (qPCR).

**Results:**

Twenty-six processed food samples were positive for the P35S alone and eight samples for the Tnos and P35S. Absolute qPCR results indicated that eleven samples were positive for GTS 40-3-2 specific event and two for MON810 specific event. A total of nine samples for events GTS 40-3-2 and MON810 exceeded the umbral allowed of transgenic content in the whole food product with the specific events. Different food products may require different DNA extraction protocols for GMO detection through PCR. Among the three methods tested, the DNeasy *mericon* food kit DNA extraction method obtained higher proportion of amplified endogenous genes through PCR. Finally, event-specific GMOs were detected in food products in Ecuador.

**Electronic supplementary material:**

The online version of this article (10.1186/s13104-017-3083-x) contains supplementary material, which is available to authorized users.

## Introduction

Biosafety regulations have been established in several countries for the proper use of genetically modified (GM) crops. The European Parliament and the corresponding member states are constantly regulating the use of GMO [1]. Few countries in the EU are growing GM crops. For instance, maize is cultivated in Spain, Portugal, Czech Republic, Romania, and Slovakia, while in others the cultivation is not established but the products of GM crops are approved for food and feed consumption [2, 3]. According to the EC Regulations No. 1829/2003 and No. 1830/2003, a content of 0.9% or above in the ingredient containing GMO have to be labelled [4].

In Ecuador, since 2000 the food products should indicate a special information in its labels if the food contains ingredients with genetic modifications according to Consumer Defense Law in its Art. 13. In 2006, the article 151 of the Ecuadorian Organic Health Law indicates that all transgenic components present in food need to be labeled. In 2009, the article 26 of the Organic Law of Food Sovereignty mentioned all the food products based on transgenic ingredients should labeled. Finally, in Art. 22 of Sanitary Regulations Substitutive Labeling of Foods Processed for Human Consumption in Ecuador in 2014 mentioned: all processed food for human consumption that contains transgenic components should include in its label the words “contains transgenic” and should be labeled if at least 0.9% of transgenic component is encountered in the whole product, not only the ingredient [5].

## Main text

### Materials and methods

#### Certified reference material

Certified reference material (CRM) from the Institute for Reference Materials and Measurements (IRMM) were used for standard curve generation in qPCR analysis for the detection of the transgenic content in food samples with soy or maize for the specific events GTS 40-3-2 and MON810, respectively (Additional file 1). DNA extraction from reference materials were performed with the commercial kit Wizard^®^ Magnetic DNA Purification System for Food (Ref: FF3750) with 100 mg of sample. The DNA was quantified (NanoDrop™ 2000, Thermo Scientific™) and diluted in a final concentration of 20 ng/µl. Then, serials dilutions were made combining the DNA from BF410gk and BF410ak (reference material GTS 40-3-2 event with 10 and 0%, respectively); independently, dilutions were made using DNA from BF413gk and BF413ak from reference material YieldGard™ event (MON810 event with 10 and 0% respectively) for the standards curve (0.625, 1.25, 2.5, 5 and 10%). The DNA extracted from Bf410ak (< 0.07%) and Bf413ak (< 0.09%) were used as negative controls.

#### Food sampling

Thirty-five processed food with soy and maize content from different branches were collected and analyzed from different Ecuadorian supermarkets, by the National Agency of Regulation Health Control and Surveillance (ARCSA) of Ecuador. The samples were classified in five different food groups to determine differences in the efficiency from the DNA extraction (Additional file 2).

#### DNA extractions and quantification from processed food samples

All samples were ground with the grinder MM400 (Retsch) at room temperature. One gram of ground sample was used for every food group as starting material for the commercial kit “Wizard^®^ Magnetic DNA Purification System for Food” (PROMEGA, Cat. FF3751) and “DNeasy *mericon* food kit” (QIAGEN, Cat. 69514), except the sausage group samples which was used 200 mg. For the conventional method of DNA extraction “CTAB” [CTAB (20 g/l), 1.4 M NaCl, 0.1 M Tris base/HCl, 20 mM Na2-EDTA (pH 8.0)], 100 mg for all samples groups was used. This conventional method is recommended by the Joint Research Centre of the European Commission [6]. The three DNA extraction methods were compared and tested in duplicates with each processed food sample and one blank that consisted in a DNA extraction without sample. For all methods used, the DNA was resuspended with nuclease free water (50–100 µl). DNA was quantified with NanoDrop™ 2000 (Thermo Scientific™), diluted to 20 ng/µl, and stored at − 20 °C until PCR reactions were performed. Statistical analysis was performed using the software InfoStat (2017. Grupo InfoStat, FCA, Universidad Nacional de Córdoba, Argentina, http://www.infostat.com.ar). For difference determination between the DNA extraction methods in the A260/A280 absorbance ratio, an ANOVA in combination with Tukey test was performed independently for each group food. A probability of *p* < 0.05 was considered to indicate significant differences. DNA quality and purity was determined through the following methods: (i) absorbance ratio A260/A280, (ii) DNA visualized by gel electrophoresis, and (iii) endogenous gene PCR amplification.

#### Primers and probes

All the primer sequences, TaqMan™ probes, and PCR conditions for endogenous genes, Cauliflower mosaic virus 35S promoter (P35S) and nopaline synthase terminator from *Agrobacterium tumefaciens* (Tnos) are described in Additional files 3 and 4, respectively [7–9].

#### Qualitative and quantitative conditions of PCR

Qualitative PCR was performed on a Mastercycler ep Gradient S No. 5345 (Eppendorf). The PCR mix had 25 µl as final volume which contained 1X-GoTaq^®^ Green Master Mix (Cat. # M7123, Promega), 0.5 µM of each primer, 2 µl of DNA template (20 ng/µl), and completed to 25 µl with nuclease-free water.

For quantitative PCR a Mastercycler^®^ ep realplex 4 No. 6302 (Eppendorf) was used to perform absolute qPCR (monoplex) for the GTS 40-3-2 and MON810 events. Each qPCR reaction mix contained 25 µl total volume with 12.5 µl of TaqMan^®^ Universal PCR Master Mix (Cat. # 4318157, Applied Biosystems); 0.5 µM and 0.3 µM of each primer for GTS 40-3-2 and MON810 events respectively; 0.2 µM of TaqMan™ probe, and completed to 25 µl with nuclease-free water. Each DNA sample was performed in triplicates in a MicroAmp^®^ Optical 96-Well Reaction Plate (Ref. # N8010560, Applied Biosytems).

The analyzes for the absolute qPCR (percentage quantification of GMO content in whole product) was performed with an interpolation of the cycle threshold (CT) mean of the unknown sample with the dilutions points of the standard curve of the CRM from the specific transgenic event, as calculated by the software of the Mastercycler^®^ ep realplex 4. Ecuador regulations for GMO labelling in food indicates that at least should be 0.9% of GMO content in the whole product (see above). Therefore, the percentage of GMO content was calculated considering only the event specific and not the endogenous gene.

#### Agarose gel electrophoresis

For the detection of amplicons for end-point PCR, and evaluation for the integrity of total DNA, agarose gel electrophoresis was performed at a 1.8 and 1% (w/v) agarose concentration, respectively. The gels were stained with SYBR™ Safe DNA Gel Stain (Cat. # S33102, Invitrogen). After electrophoresis the gels were visualized using the Gel Doc XR+ (BIO-RAD) with the Quantity One software.

### Results and discussion

#### DNA extraction

**Fig. 1:**
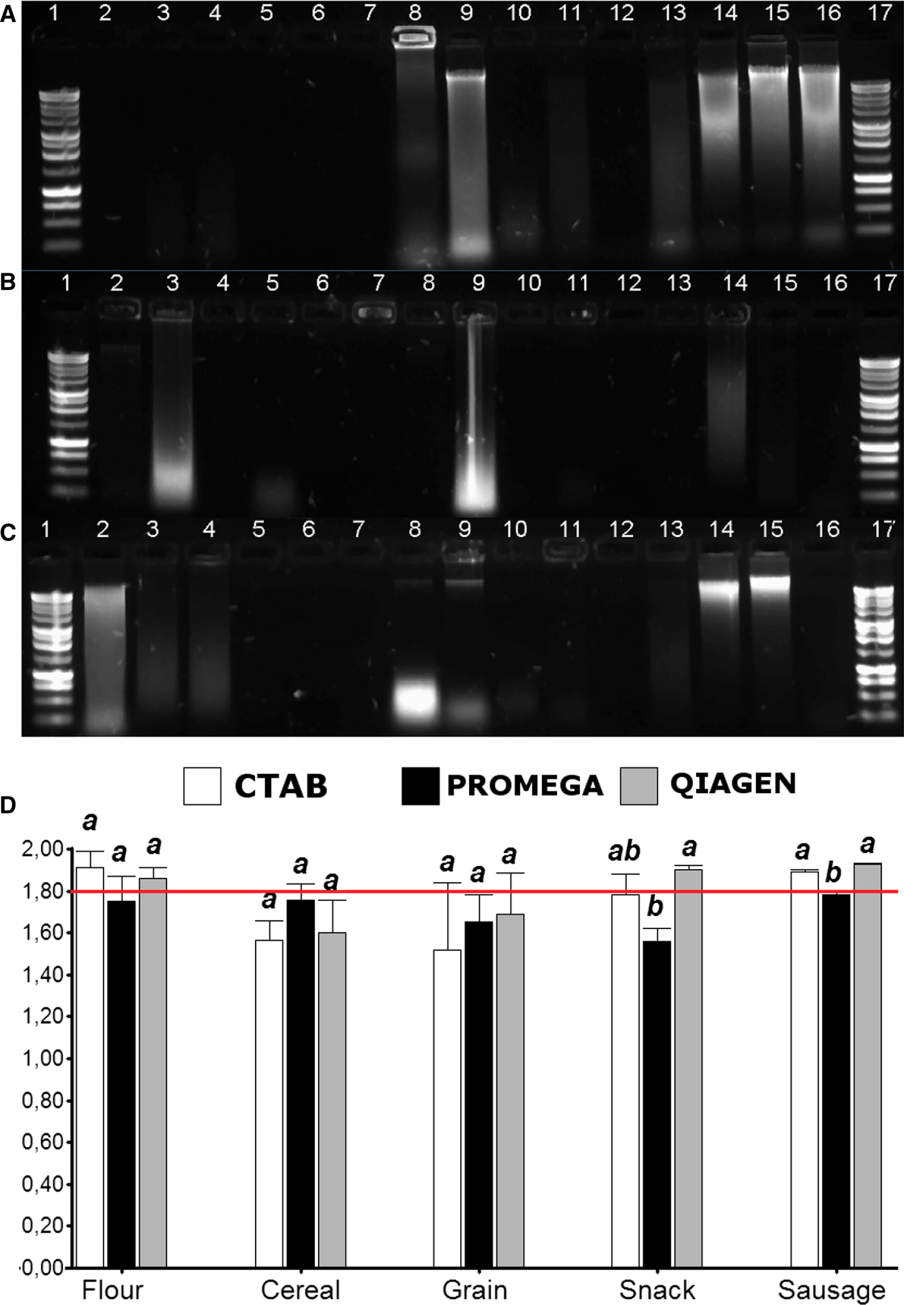
DNA extraction methods. **A** CTAB [6], **B** Wizard^®^ Magnetic DNA Purification System for Food (PROMEGA, REF: FF3751), **C** DNeasy *mericon* food (QIAGEN, Cat 69514). Lanes 1 and 17: 1 Kb Ladder (PROMEGA, REF: G571A), lanes 2–4 (flour): DNA extracted from banana and soy flour, pre-cooked maize flour. Lanes 5–7 (cereal): DNA extracted from oats, soy, and flakes. Lanes 8–10 (grain): DNA extracted from soy grain, microwave popcorn, and sweet corn. Lanes 11–13 (snack): DNA extracted from corn chips, cheese, and corn snacks. Lanes 14–16 (sausage): DNA extracted from pig, res, species sausages. The lanes were loaded with 5 µl of DNA samples. **D** A260/280 ratio from DNA extracted with CTAB method [6], Wizard^®^ Magnetic DNA Purification System for Food (PROMEGA, REF: FF3751), and DNeasy *mericon* food (QIAGEN, Cat. 69514), measured with NanoDrop™ 2000 (Thermo Scientific). Variance analysis using ANOVA in combination with Tukey test show significant differences within the DNA extraction methods in the snack and sausage group food. Values with the same letter are not statistically different (p > 0.05). Tukey test was made independently for each group food. The bars represent the standard error from the average (flour n = 10; cereal n = 4; grain n = 3; snack n = 4; sausage n = 14). The red line at 1.8 indicate the value optimum for 260/280 ratio

Three methodologies for DNA extraction in processed food were compared. The CTAB conventional methodology use chemical agents and organic solvents for the purification phase. Some commercials kits use magnetic bead that binds the DNA in a saline solution (Wizard^®^ Magnetic, Promega), and others use columns with silica membrane (DNeasy, Qiagen). Manufacturing food products influence the quality and quantity of the DNA extracted. The DNA extracted may have PCR inhibitors including lipids, polyphenols, and polysaccharides, avoiding amplification by PCR. Therefore, the extracted DNA from some samples showed a low concentration and the DNA could not be visible in an agarose gel (Fig. 1A) [10].

The methods showing a greater amount of genomic DNA were the CTAB and DNeasy *mericon* food kit in sausage and grains groups, whereas the other groups showed degradation in the DNA, and may present few contaminant factors that inhibit the DNA extraction, which are difficult to remove [11]. Besides, degraded DNA could be explained by the type of processing in the product elaboration, which may use strong alkaline or acid agents that produce hydrolytic degradation of DNA; furthermore, the exposing for long periods at high temperatures resulted in fragmentation of high molecular weight DNA which consequently affects the PCR analysis [12, 13]. Significant differences of DNA quality measured by absorbance (A260/A280) was encountered within snack and sausage groups. The DNA extracted using the CTAB and DNeasy *mericon* food kit protocols were close to the optimum ratio of 1.8 (Fig. 1D) [14]. Combination of protocols and/or commercial kits could be performed to increase the DNA yield and quality.

#### Detection of *lectin* and *alcohol dehydrogenase* (*adh*) genes

PCR of endogenous genes was performed on the DNA extracted (Table 1). The results showed that all methods evaluated may either had PCR inhibitors or the DNA extracted is highly degraded, because in many samples the expected amplicon from the endogenous genes were not amplified. The DNeasy *mericon* food kit showed the greater amount of amplicons in all samples food groups compared with CTAB and Wizard^®^ methods (Additional file 5). Considering that purity and integrity are important factors to get consistent and accurate results, DNA with optimum qualities from all methods evaluated were selected for GMO detection [15]. Furthermore, depending of the trace amount from some ingredients a trace GMO ingredient could be detected [16].

**Table 1:** GMO detection of food products according to group type

Food groups	Code	Number of samples	Endogenous genes		Endogenous genes detection^a^			Transgenic event detection						
					Conventional method	Commercial kits		Qualitative PCR P35S and t-NOS^b^			Percentage of GTS 40-3-2 event in whole food product^c^		Percentage of MON810 in whole food product^c^	
					CTAB	Wizard^®^ Magnetic DNA Purification System for Food (PROMEGA)	Dneasy *mericon* food (QIAGEN)	P35S positive	Tnos positive	P35S and Tnos positive	Code group	%	Code group	%
Flour	F	10	*Lectin*		5	5	7	9	5	5	F1	0.1	F2	0.1
											F3	1.8		
			*Alcohol dehydrogenase*		4	5	4				F6	4.7	F6	3.7
											F8	2.3		
Cereal	C	4	*Lectin*		2	1	3	4	0	0	C1	1.1		
			*Alcohol dehydrogenase*		1	0	1							
Grain	G	3	*Lectin*		1	1	3	3	0	0	G3	0.1		
			*Alcohol dehydrogenase*		2	2	1							
Snacks	Sk	4	*Lectin*		0	0	3	4	1	1	n/a	n/a		
			*Alcohol dehydrogenase*		2	4	1				n/a	n/a		
Sausage	S	14	*Lectin*		11	9	14	6	2	2	S1	2.3		
											S2	2.1		
			*Alcohol dehydrogenase*		0	3	1				S3	4.2		
											S5	2.3		
											S13	1.9		
Total		35						26	8	8				

^a^PCR amplification of lectin and alcohol dehydrogenase genes

^b^P35S refers to 35S promoter from the Cauliflower mosaic virus and Tnos refers to nopaline synthase terminator from *Agrobacterium tumefaciens*

^c^GTS 40-3-2 and MON810 quantification with qPCR

The CTAB conventional method use organic solvents in the purification phase; therefore, PCR inhibitors could be encountered in the extracted DNA. On the other hand, depending of the food sample, amplification by PCR is halted due to inhibitors. Commercial kits for DNA extraction of food could be efficient; however, depending of the food group, the DNA extracted is minimal and with some impurities [17, 18]. Therefore, for GMO detection, the materials used for the manufacturing of the food could be analyzed instead of the final product. Each food group would have an optimum DNA extraction method.

#### GMO screen P35S/Tnos

DNA showing endogenous gene amplification from the three methods were selected to detect the presence of P35S and Tnos. Twenty-six samples were positive for the P35S and only eight for Tnos from a 35 samples analyzed in total, respectively (Table 1 and Additional file 5). This proportion of GMO in food indicate that most of the soy and maize used to manufacture food in Ecuador might be imported from GM growing countries including USA and Argentina [19, 20]. From the total of samples analyzed, eight were from imported products and 27 from national manufacturers. Information regarding GMO events present in food ingredients imported from GM crops cultivated internationally should be available to detect the presence of specific events.

#### GTS 40-3-2 and MON810 detection and quantification from processed food samples

According to ISAAA, a total of 230 transgenic events are approved for maize and 36 transgenic events for soy. The GTS 40-3-2 and MON810 events are some of the most cultivated worldwide [21]. Each event was analyzed through an absolute qPCR to quantify event content.

Twenty-six samples that were positive for P35S and Tnos revealed that eleven samples were positive for GTS 40-3-2 transgenic event, and only two samples were below the label threshold of 0.9% while nine samples contained > 0.9%. Besides, only two samples resulted positive for MON810 and one sample contains below 0.9%. These positive MON810 food product were positive also for GTS 40-3-2 (Table 1). A total of nine samples between GTS 40-3-2 and MON810 exceeded the umbral allowed of transgenic component to be labeled, and only four food products were labeled with “contains transgenic” as indicated in article 151 of the Ecuadorian Organic Health Law [5].

### Conclusion

DNA quantity and quality depends of the type of food and the extraction method. The DNeasy *mericon* food kit method showed better results in the integrity and quality with the sausage food group. Furthermore, results showed that DNeasy *mericon* food kit obtained the greater number of samples with amplicons of endogenous genes from soy and maize in all food groups. Besides, GMO screen for determination of the P35S and Tnos clearly demonstrated the presence of GM in processed food products. Some products were above the threshold (0.9%) for labelling but not all were labeled. Information related to the traceability of GMO events present in imported food ingredients should be available for GMO detection laboratories to determinate the presence of specific events.

## Limitations

Main limitations include: (i) low DNA qualities after extraction from processed food which hampered the GMO detection; and, (ii) lack of databases indicating transgenic events in processed food distributed in Ecuador.

## Additional files

**Additional file 1.** Certified Reference Material used for positive/negative controls and for GMO content quantification. Certified Reference Material from specific transgenic events.

**Additional file 2.** Food groups samples. Food groups samples with the code and the number of samples in each group.

**Additional file 3.** Primers and probes sequences. Primers and probes sequences used for the GMO detection and quantification.

**Additional file 4.** Qualitative and Quantitative PCR conditions. PCR conditions used for the GMO detection and quantification.

**Additional file 5.** Qualitative PCR. Qualitative PCR results in gel electrophoresis.

## Abbreviations

*adh*: alcohol dehydrogenase gene
ANOVA: analysis of variance
ARCSA: National Agency of Regulation Health Control and Surveillance
CRM: certified reference material
CTAB: cetyltrimethylammonium bromide
DNA: deoxyribonucleic acid
GM: genetically modified
GMOs: genetically modified organisms
IRMM: Institute for Reference Materials and Measurements
ISAAA: International Service for the Acquisition of Agri-biotech Applications
P35S: Cauliflower mosaic virus 35S-promoter
PCR: polymerase chain reaction
qPCR: quantitative PCR
Tnos: nopaline synthase-terminator from *Agrobacterium tumefaciens*
