# Supplementary material for: Comparison of three DNA extraction methods for the detection and quantification of GMO in Ecuadorian manufactured food
Source: BMC Res Notes. 2017 Dec 20;10:758. doi: 10.1186/s13104-017-3083-x (PMC5738804; doi:10.1186/s13104-017-3083-x)
Supplement: Supplementary file 1 — Additional file 1. Certified Reference Material used for positive/negative controls and for GMO content quantification. Certified Reference Material from specific transgenic events. [file 13104_2017_3083_MOESM1_ESM.docx]

**Additional file 1**

**Certified Reference Material used for positive/negative controls and for GMO content quantification**.

| CIBE^a^ CODE | CRM^b^ CODE | EVENT | PERCENTAGE (%) |
| --- | --- | --- | --- |
| ERM 001 | BF410ak | GTS 40-3-2 | 0 |
| ERM 004 | BF413aK | MON810 | 0 |
| ERM 006 | BF410gk | GTS 40-3-2 | 10 |
| ERM 009 | BF413gK | MON810 | 10 |

^a^Biotechnology Research Center of Ecuador (Spanish acronym for “Centro de Investigaciones Biotecnológicas del Ecuador”).

^b^Certified Reference Material.
