# Supplementary material for: Comparison of three DNA extraction methods for the detection and quantification of GMO in Ecuadorian manufactured food
Source: BMC Res Notes. 2017 Dec 20;10:758. doi: 10.1186/s13104-017-3083-x (PMC5738804; doi:10.1186/s13104-017-3083-x)
Supplement: Supplementary file 2 — Additional file 2. Food groups samples. Food groups samples with the code and the number of samples in each group. [file 13104_2017_3083_MOESM2_ESM.docx]

**Additional file 2**

**Food groups samples.**

| Code | Food group | Number of food products |
| --- | --- | --- |
| F | Flour | 10 |
| C | Cereal | 4 |
| G | Grain | 3 |
| Sk | Snack | 4 |
| S | Sausage | 14 |
|  | TOTAL | 35 |
