# Supplementary material for: Comparison of three DNA extraction methods for the detection and quantification of GMO in Ecuadorian manufactured food
Source: BMC Res Notes. 2017 Dec 20;10:758. doi: 10.1186/s13104-017-3083-x (PMC5738804; doi:10.1186/s13104-017-3083-x)
Supplement: Supplementary file 3 — Additional file 3. Primers and probes sequences. Primers and probes sequences used for the GMO detection and quantification. [file 13104_2017_3083_MOESM3_ESM.docx]

**Additional file 3**

**Primers and probes sequences**

| **Primer** | **Sequence** | **Target element** | **Fragment length** | **PCR type** | **Reference** | **Supplier** |
| --- | --- | --- | --- | --- | --- | --- |
| Le1n02-5/Le1n02-3 | 5'-GCCCTCTACTCCACCCCCA-3' | Soy lectin gen | 118 | Qualitative | [7] | eurofins Genomics |
|  | 5-'GCCCATCTGCAAGCCTTTTT-3' |  |  |  |  |  |
| Adh-F3/Adh-R1 | 5-CGTCGTTTCCCATCTCTTCCTCC-3' | Maize alcohol deshidrogenase (*adh*) gen | 231 | Qualitative | [7] | eurofins Genomics |
|  | 5-GACAGAGGAGAAACAAGGCG-3' |  |  |  |  |  |
| P35s-CF3/P35s-CR4 | 5'-CCA CGTCTTCAAAGCAAGTGG-3' | 35S promoter | 123 | Qualitative | [8] | eurofins Genomics |
|  | 5'-TCCTCTCCAAATGAAATGAACTTCC-3' |  |  |  |  |  |
| HA-nos118-f/HA-nos118-r | 5'-GCATGACGTTATTTATGAGATGGG-3' | Nos-terminator | 118 | Qualitative | [9] | eurofins Genomics |
|  | 5'-GACACCGCGCGCGATAATTTATCC-3' |  |  |  |  |  |
| RRS01-5’/RRS 01-3’ | 5'-CCTTTAGGATTTCAGCATCAGTGG-3' | Event -GTS-40-3-2 | 121 | Quantitative | [7] | eurofins Genomics |
|  | 5'-GACTTGTCGCCGGGAATG-3' |  |  |  |  |  |
| RRS-Taq | 5' -6-FAM-CGCAACCGCCCGCAAATCC-TAMRA -3' | probe |  |  |  | Applied Biosystems |
| MON810F/MON810R | 5'-TCGAAGGACGAAGGACTCTAACGT-3' | Event-MON810 | 92 | Quantitative | [7] | eurofins Genomics |
|  | 5'-GCCACCTTCCTTTTCCACTATCTT-3' |  |  |  |  |  |
| MON810-Taq | 5' -6-FAM-AACATCCTTTGCCATTGCCCAGC-TAMRA -3' | probe |  |  |  | Applied Biosystems |
