# Supplementary material for: Comparison of three DNA extraction methods for the detection and quantification of GMO in Ecuadorian manufactured food
Source: BMC Res Notes. 2017 Dec 20;10:758. doi: 10.1186/s13104-017-3083-x (PMC5738804; doi:10.1186/s13104-017-3083-x)
Supplement: Supplementary file 4 — Additional file 4. Qualitative and Quantitative PCR conditions. PCR conditions used for the GMO detection and quantification. [file 13104_2017_3083_MOESM4_ESM.docx]

**Additional file 4.**

**Qualitative and Quantitative PCR conditions**.

| **Qualitative PCR** | | | | | | |
| --- | --- | --- | --- | --- | --- | --- |
| **Primer pair** | **Initial denaturation** | **Denaturation** | **Annealing** | **Extension** | **Cycles** | **Final elongation** |
| Le1n02-5/Le1n02-3 | 5 min at 95°C | 30 s at 95°C | 30 s at 60°C | 30 s at 72°C | 37 | 5 min at 72°C |
| Adh-F3/Adh-R1 | 5 min at 95°C | 30 s at 95°C | 30 s at 60°C | 30 s at 72°C | 37 | 5 min at 72°C |
| P35s-CF3/P35s-CR4 | 5 min at 95°C | 30 s at 95°C | 30 s at 62°C | 45 s at 72°C | 50 | 7 min at 72°C |
| HA-nos118-f/HA-nos118-r | 5 min at 95°C | 30 s at 95°C | 30 s at 62°C | 45 s at 72°C | 50 | 7 min at 72°C |
| **Absolute Quantitative PCR/TaqMan probe** | | | | | | |
| **Primer pair** | **UDG** | **Initial denaturation** | **Denaturation** | **Annealing/Elongation** | **Cycles** |  |
| RRS 01-5/RRS 01-3 | 2 min at 50°C | 95°C for 10 min | 95°C for 30 seg | 60°C for 1 min | 50 |  |
| MON810F/MON810R | 2 min at 50°C | 95°C for 10 min | 95°C for 15 seg | 60°C for 1 min | 50 |  |
