# Supplementary material for: Comparison of three DNA extraction methods for the detection and quantification of GMO in Ecuadorian manufactured food
Source: BMC Res Notes. 2017 Dec 20;10:758. doi: 10.1186/s13104-017-3083-x (PMC5738804; doi:10.1186/s13104-017-3083-x)
Supplement: Supplementary file 5 — Additional file 5. Qualitative PCR. Qualitative PCR results in gel electrophoresis. [file 13104_2017_3083_MOESM5_ESM.docx]

**Additional file 5**

**Qualitative PCR
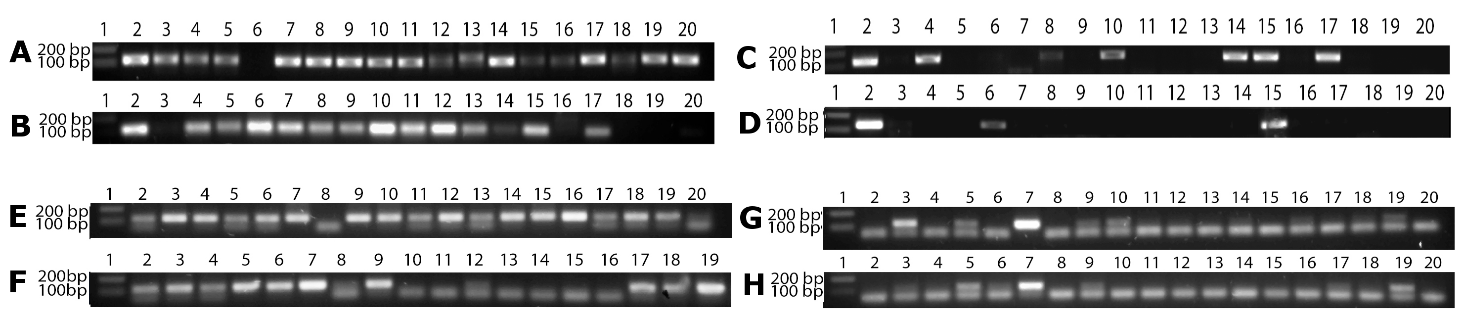
**

**A-** Detection of *lectin* gene (118 bp) from soy in DNA extracted with the DNeasy *mericon* Food (QIAGEN). Lane 1: 100 bp ladder (PROMEGA, Cat# 62101). Lane 2: Positive control with DNA from referential flour “BF410gk”. Lanes 3 to 6 (cereals): DNA extracted from oats and soy cereal, flakes cereal. Lanes 7 to 20: (sausages): DNA extracted from pig, res, species sausages. **B-** Lectin amplification. Lane 1: 100 bp ladder (PROMEGA, Cat# 62101). Lane 2: positive control with DNA from referential flour “BF410gk”. Lanes 3 and 20: negative control with water. Lanes 4 to 6 (Snacks): DNA extracted from corn chips, cheese and corn snacks. Lanes 7 to 9 (Grains): DNA extracted from soy grain, microwave popcorn, sweet corn. Lanes 10 to 19 (Flours): DNA extracted from banana and soy flour, pre-cooked maize flour. Each sample food group have different brand products. **C-** Detection of the *alcohol dehydrogenase* (*adh*, 231 bp) gene in DNA extracted with the DNeasy *mericon* Food kit (QIAGEN). Lane 1: 100 bp ladder (PROMEGA, Cat# 62101). Lane 2: positive control with DNA from referential flour “BF413gK”. Lanes 3 to 5 (Snacks): DNA extracted from corn chips, cheese and corn snacks. Lanes 6 to 8 (Grains): DNA extracted from soy grain, microwave popcorn, sweet corn. Lanes 9 to 18 (Flours): DNA extracted from banana and soy flour, pre-cooked maize flour. Lanes 19 to 20: negative control with water. **D-** *adh* amplification in DNA extracted with the DNeasy *mericon* Food kit (QIAGEN). Lane 1: 100 bp ladder (PROMEGA, Cat# 62101). Lane 2: Positive control with DNA from referential flour “BF413gK”. Lanes 3 to 6 (Cereals): DNA extracted from oats and soy cereal, flakes cereal. Lanes 7 to 20 (Sausages): DNA extracted from pig, res, species sausages. Each sample food group have different brand products. **E-** GMO screening for the presence of the P35S (123 bp) from processed food samples. Lane 1: 100 bp ladder (PROMEGA, Cat# 62101). Lanes 2 to11 (Flours): DNA extracted from banana and soy flour, pre-cooked maize flour. Lanes 12 to 15 (Cereals): DNA extracted from oats and soy cereal, flakes cereal. Lanes 16 to 18 (Grains): DNA extracted from soy grain, microwave popcorn, sweet corn. Lane 19 (Snack): DNA extracted from corn chips. Lane 20: Negative control with DNA from referential flour “BF410ak”. **F-** P35S amplification. Lane 1: 100 bp ladder (PROMEGA, Cat# 62101). Lanes 2 to 4 (Snacks): DNA extracted from corn chips, cheese and corn snacks. Lane 5 to 18 (Sausages): DNA extracted from pig, res, species sausages. Lane 19: Positive control with DNA from referential flour “BF410gK”. Each samples food group have different brand products. Most PCR products showed primers dimers. **G**- GMO screening for the presence of Tnos (118 bp) from processed food samples. Lane 1: 100 bp ladder (PROMEGA, Cat# 62101). Lanes 2 to 11 (Flours): DNA extracted from banana and soy flour, pre-cooked maize flour. Lanes 12 to 15 (Cereals): DNA extracted from oats and soy cereal, flakes cereal. Lanes 16 to 18 (Grains): DNA extracted from soy grain, microwave popcorn, sweet corn. Lane 19 (Snack): DNA extracted from corn chips. Lane 20: Negative control with DNA from referential flour “BF410ak”. **H-** Tnos amplification. Lane 1: 100 bp ladder (PROMEGA, Cat# 62101). Lanes 2 to 4 (Snacks): DNA extracted from corn chips, cheese and corn snacks, lane 5 to 18 (Sausages): DNA extracted from pig, res, species sausages. Lane 19: Positive control with DNA from referential flour “BF410gK”. Lane 20: Negative control with DNA from referential flour “BF410ak”. Each sample food group have different brand products. Most PCR reactions showed primers dimers (<100 bp).
